# Identification and Validation of Telomere-Related Gene Signature in Intervertebral Disc Degeneration

**DOI:** 10.7759/cureus.71735

**Published:** 2024-10-17

**Authors:** Shiwei Xie, Heng Xiao, Fan Zhang, YuPing Lan, Mingwei Luo

**Affiliations:** 1 Orthopedics, Panzhihua Central Hospital, Panzhihua, CHN; 2 Orthopedics, The First Affiliated Hospital of Kunming Medical University, Kunming, CHN

**Keywords:** bioinformatics analysis, differential expression, hub genes, intervertebral disc degeneration, telomere-related genes

## Abstract

This study investigates the role of telomere-related differentially expressed genes (TRDEGs) in intervertebral disc degeneration (IVDD) through comprehensive bioinformatics analyses. Data were sourced from the Gene Expression Omnibus (GEO) with datasets GSE245147 and GSE124272 used for initial identification and validation, respectively. The GSE245147 dataset comprised transcriptional profiles from nucleus pulposus cells of both degenerated and non-degenerated human nucleus pulposus (NP) tissues. Using the limma package, 198TRDEGs were identified by intersecting differentially expressed genes (DEGs) with telomere-related genes (TRGs) from the TelNet database. Functional enrichment analyses using the Database for Annotation, Visualization and Integrated Discovery (DAVID) revealed that TRDEGs are significantly involved in cell division, chromosome segregation, and other mitotic processes. Protein-protein interaction (PPI) networks constructed using the Search Tool for the Retrieval of Interacting Genes/Proteins (STRING) database and visualized with Cytoscape (Cytoscape Consortium, San Diego, CA, USA) identified key hub genes such as CDK1, CCNA2, and AURKB. Pearson correlation and receiver operating characteristic (ROC) analyses highlighted five hub genes (ASPM, BUB1B, CDC20, KIF2C, TTK) with significant predictive value for IVDD. Additionally, mRNA-microRNA (miRNA) interaction analysis using NetworkAnalyst identified key miRNAs interacting with these hub genes. This study provides insights into the molecular mechanisms of IVDD and identifies potential targets for therapeutic intervention.

## Introduction

Cervical spondylosis [1] is a degenerative disease that represents the most common spinal column disorder in adults. The main clinical symptoms of cervical spondylosis include pain in the neck, shoulder, and back, and numbness and weakness of the limbs, which not only cause the loss of individual labor but also results in the consumption of a large amount of healthcare resources and costs; it is a huge economic burden [2] to many families and society. Cervical intervertebral disc degeneration (CIDD) is a major factor in the development of this disease, but the etiology and pathological mechanisms of CIDD are not fully understood. Therefore, at present, the diagnosis and treatment of CIDD and cervical spondylosis mainly rely on clinical signs, imaging, surgery, and physical therapy [3]. It is difficult to use biochemical indicators to accurately measure the degree of CIDD as well as to carry out precise molecular therapy. Therefore, there is a great need for research to establish biochemical indicators for CIDD and to find effective molecular therapeutic targets based on these indicators to lay the foundation for future diagnosis and treatment. Recent studies [4,5] have found that CIDD is often aggravated by the aging process in the human body, and it is believed that aging is the main factor leading to cervical CIDD and ultimately causing cervical spondylosis.

In recent years, a study [6] has shown that telomere length can be used as an extremely important indicator of cellular senescence. Telomeres are repetitive sequences of DNA at the ends of chromosomes (e.g., the human telomere repeats are TTAGGG), and their main role is to maintain the integrity of chromosomes [7]. Telomere length varies widely among species; for example, the length of telomere in different cells of a human is about 2 kb to 15 kb, while the length of telomere in different cells of a mouse is about 20 kb to 150 kb [7]. Telomere length is closely associated with processes such as autoimmune diseases, aging, inflammation, cell differentiation, tissue regeneration, and the development of malignant tumors [8] in humans. Overall, telomere length becomes progressively shorter with age and is often accompanied by several diseases. Numerous factors affect telomere length, such as an unhealthy lifestyle including smoking, drinking alcohol, eating unhealthy food, and not participating in exercise, which can accelerate the process of telomere length shortening [9]. At the protein level, mutations, loss of function, or altered activity of DNA polymerase, telomerase, and shelterin protein complex, which are required for telomeric DNA replication, may lead to telomere length abnormalities in cells. In yeast cells, it has been shown that [10] abnormalities in the function of three polymerases involved in telomeric DNA replication can lead to alterations in cellular telomere length.

Telomere-related genes (TRGs), such as telomerase reverse transcriptase (TERT) and telomerase RNA component (TERC), are essential [11] for maintaining telomere length and function. Mutations or dysregulation in these genes can lead to a variety of diseases, including cancer and premature aging syndromes. Recent studies have also found a link between telomere shortening and disc degeneration. Telomere shortening in disc cells [12] can lead to reduced cellular function, accelerating the process of disc degeneration. Understanding the mechanisms of telomere biology and its role in disc degeneration is crucial for developing therapeutic strategies to combat age-related diseases and improve the management of disc degeneration. In this study, we aim to comprehensively explore the key roles of telomeres in the pathogenesis and progression of intervertebral disc degeneration (IVDD) using mature and established bioinformatic analysis methods, with the hope of identifying novel diagnostic and therapeutic targets for IVDD.

## Materials and methods

Data collection

The data used in this study are available from the Gene Expression Omnibus (GEO) repository (https://www.ncbi.nlm.nih.gov/geo/) with accession number GSE245147 [13], which was used to identify differentially expressed genes (DEGs) associated with telomere-associated genes. For the validation analysis, the GEO dataset GSE124272 [14,15] was used. Dataset GSE254147 comprised the transcriptional profiles of nucleus pulposus (NP) cells from three degenerated and three non-degenerated human NP tissues. Dataset GSE124272 included whole blood samples from eight patients with IVDD and eight healthy controls (Table 1). Telomere-related genes, including markers, drivers, suppressors of telomere function, and unclassified genes, were obtained from the TRGs downloaded from TelNet (http://www.cancertelsys.org/telnet/). 

**Table 1:** Statistics of the two databases derived from the GEO database GEO: Gene Expression Omnibus

Serier	Degenerated	Non-degenerated	Total
GSE254147	3	3	6
GSE124272	8	8	16

Determination of differentially expressed genes (DEGs) and telomere-related DEGs 

The DEGs in IVDD were identified using the R package limma version 3.60.2 (Bioconductor, Boston, MA, USA), with criteria of |logFC| > 1 and adjusted p-value < 0.05. Illustrations of volcano plots and hierarchical cluster heat maps show the DEGs were generated using the R package 'ggplot2' version 3.5.1 (R Foundation for Statistical Computing, Vienna, AUT). The telomere-related differentially expressed genes (TRDEGs) were determined by overlapping the DEGs from the GSE245147 dataset with TRGs from the TelNet database using a Venn diagram tool (http://bioinformatics.psb.ugent.be/webtools/Venn/).

Functional enrichment analysis

Kyoto Encyclopedia of Genes and Genomes (KEGG) and Gene Ontology (GO) analyses were performed on the TRDEGs using the Database for Annotation, Visualization and Integrated Discovery (DAVID) (https://david.ncifcrf.gov/tools.jsp). The GO is an internationally standardized system for the classification of gene functions, comprising three categories: biological process (BP), cellular component (CC), and molecular function (MF). The GO analysis was performed using the R 4.4.0 package 'GOplot' version 3.5.1 (R Foundation for Statistical Computing, Vienna, AUT) with GO annotations. The KEGG analysis, aimed at identifying relevant pathways for TRDEGs, was also based on DAVID. Items with a p-value < 0.05 were considered statistically significant. The enriched items were visualized using Microsoft Excel (Microsoft Corp., Redmond, WA, USA) bar charts.

Protein-protein interaction (PPI) network analysis

The PPI network analysis of the TRDEGs was performed using the Search Tool for the Retrieval of Interacting Genes/Proteins (STRING) database version 12.0 (https://cn.string-db.org/), a widely used tool for assessing PPIs. Protein interaction pairs with a score > 0.40 were then imported into Cytoscape software version 3.7.1 (Cytoscape Consortium, San Diego, CA, USA) to construct the PPI network. In this network, nodes represent the TRDEGs identified in the STRING database, while edges indicate the interactions between these TRDEGs. The PPI score was calculated using the degree analysis method within the CytoHubba plug-in, and the top 10 nodes with the highest connectivity were identified as hub TRDEGs for further investigation.

Correlation analysis among the hub TRDEGs

To assess the relationships between these hub TRDEGs, the Pearson correlation analysis was used to examine the correlations. In this analysis, the r-value represents the correlation coefficient, which is used to assess the effect size. The correlation matrix heatmap and scatter plots were then generated using the R package 'ggplot2' version 3.5.1.

Receiver operating characteristic (ROC) analysis of the hub TRDEGs

We used ROC analysis using the R software package 'pROC' version 1.18.5 (R Foundation for Statistical Computing, Vienna, AUT) to calculate the area under the curve (AUC) values. First, gene expression data for the hub TRDEGs were extracted from the GEO dataset GSE124272. The ROC function from pROC was used for ROC analysis, while the confidence interval (CI) function was used to determine AUC and CIs. The ROC curve plots sensitivity values on the y-axis against false positive rates (1-specificity) on the x-axis. An AUC value ≥ 0.70 on the ROC curve indicated sufficient predictive ability.

The mRNA-microRNA (miRNA) interaction analysis

We screened out 10 hub genes and used the genes for mRNA-miRNA interaction analysis on NetworkAnalyst (https://www.networkanalyst.ca). The constructed network was visualized using NetworkAnalyst's visualization tool to generate graphical representations of nodes and edges. Nodes represent genes or miRNAs, and edges represent interactions between them. The constructed network was analyzed for topology, measuring the extent to which a node mediates the shortest path between other nodes in the network based on betweenness centrality, calculating key network metrics, and identifying potential key regulatory molecules.

## Results

Determination of TRDEGs

**Figure 1:**
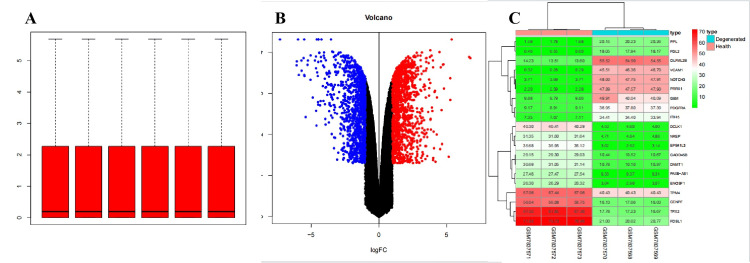
Identification of DEGs between the IVDD group and control group A: The box plot of the expression matrix of the GSE245147 dataset in the GEO database after normalization. B: Volcano map of DEGs after differential analysis of the expression matrix. The x-axis represents -log10 (p-value) and the y-axis represents log2 (conversion change). In the image, the red dots represent up-regulated genes, the blue dots represent down-regulated genes, and the black dots represent genes with no significant difference in expression. C: Thermogram of DEGs analyzed according to GSE245147 DEGs: Differentially expressed genes, IVDD: Intervertebral disc degeneration, GEO: Gene Expression Omnibus

**Figure 2:**
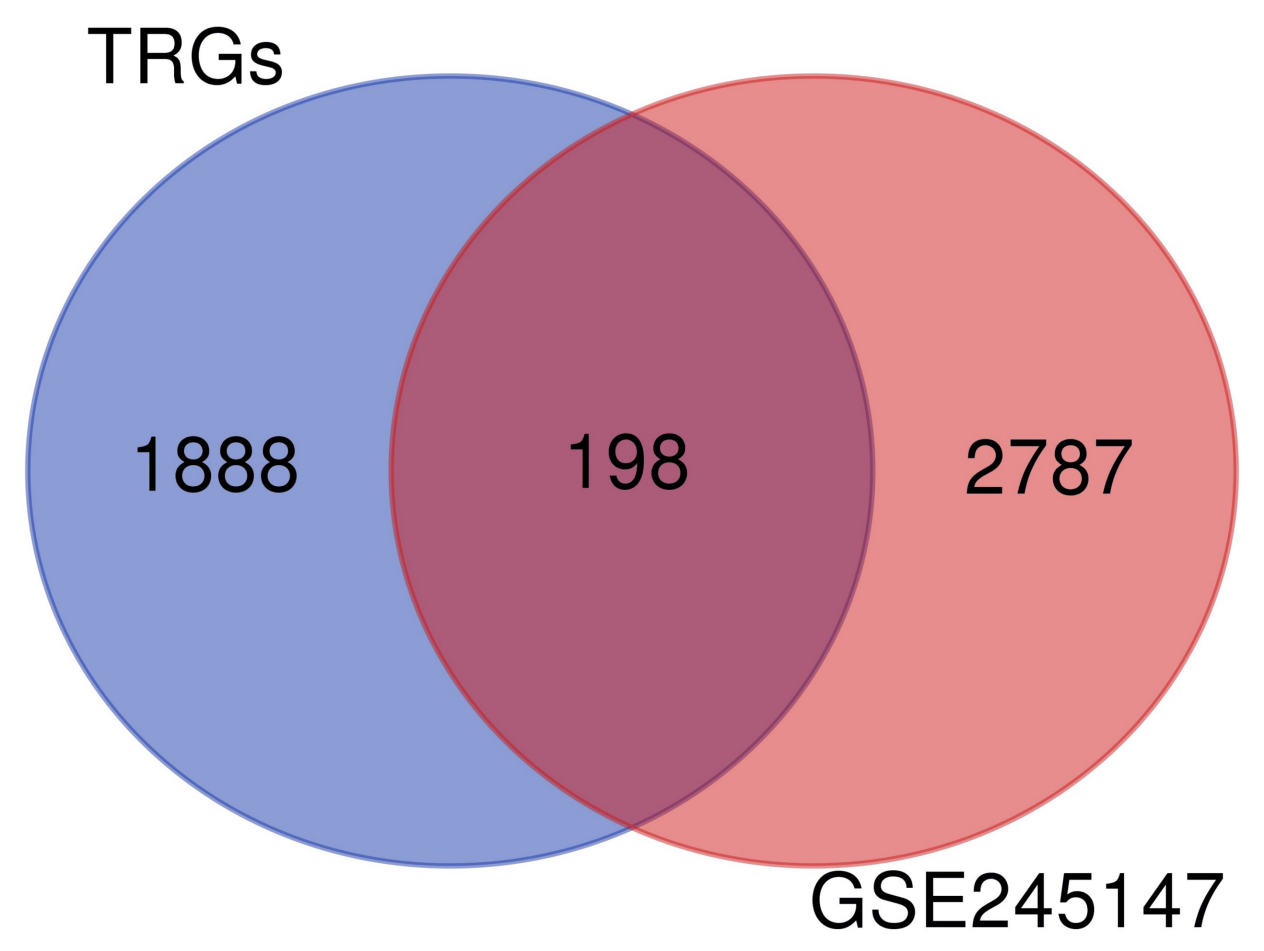
Venn diagram of the DEGs analyzed by GSE245147 and the TRGs based on TelNet DEG: Differentially expressed genes, TRGs: Telomere-related genes

Based on the GSE245147 dataset, we conducted a differential analysis between normal and degenerated samples. Using the R package limma version 3.60.2, volcano plots and heatmaps were generated. A total of 2985 DEGs were identified, comprising 1613 up-regulated and 1372 down-regulated genes (Figure 1). Next, we intersected the DEGs obtained with a set of TRGs and plotted a Venn diagram. This yielded a total of 198 TRDEGs (Figure 2). These DEGs were used for the next stage of analysis.

The GO and KEGG function analysis of the TRDEGs

**Figure 3:**
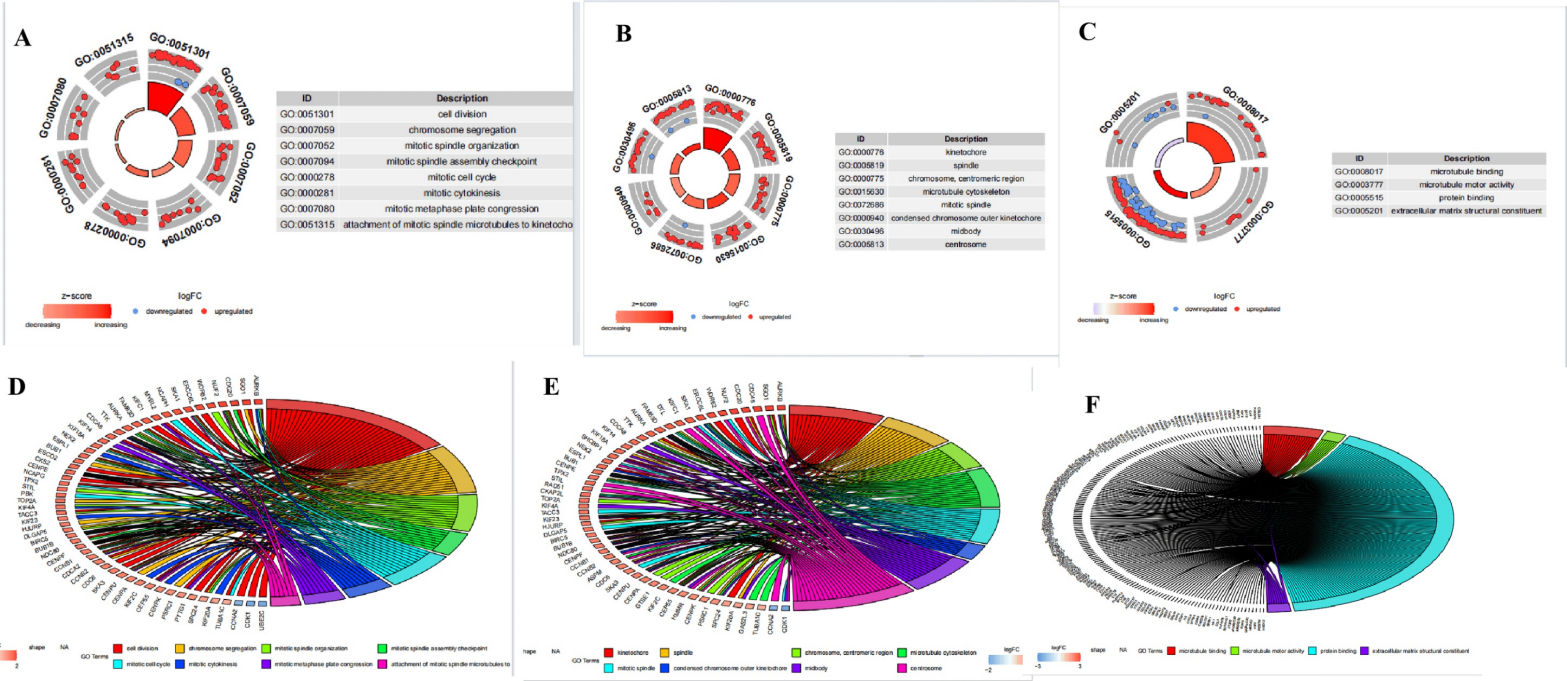
Functional enrichment analysis of TRDEGs A: The top eight significantly enriched GO terms in the category of BP for the FRDEGs; B: The top eight significantly enriched GO terms in the category of CC; C: The top four significantly enriched GO terms in the category of MF; D: The KEGG pathway enrichment analysis for FRDEGs; D: Circle plot of GO terms for the first eight apparent enrichments of these central TRDEGs in the BP category; E: Circle diagram of the top eight GO terms significantly enriched in the CC category; G: Circle diagram of the top four GO terms significantly enriched in the MF category MF TRDEGs: Telomere-related differentially expressed genes, GO: Gene Ontology, KEGG: Kyoto Encyclopedia of Genes and Genomes, FRDEGs: Ferroptosis-related differentially expressed genes, CC: Cellular component, MF: Molecular function, BP: Biological process

**Figure 4:**
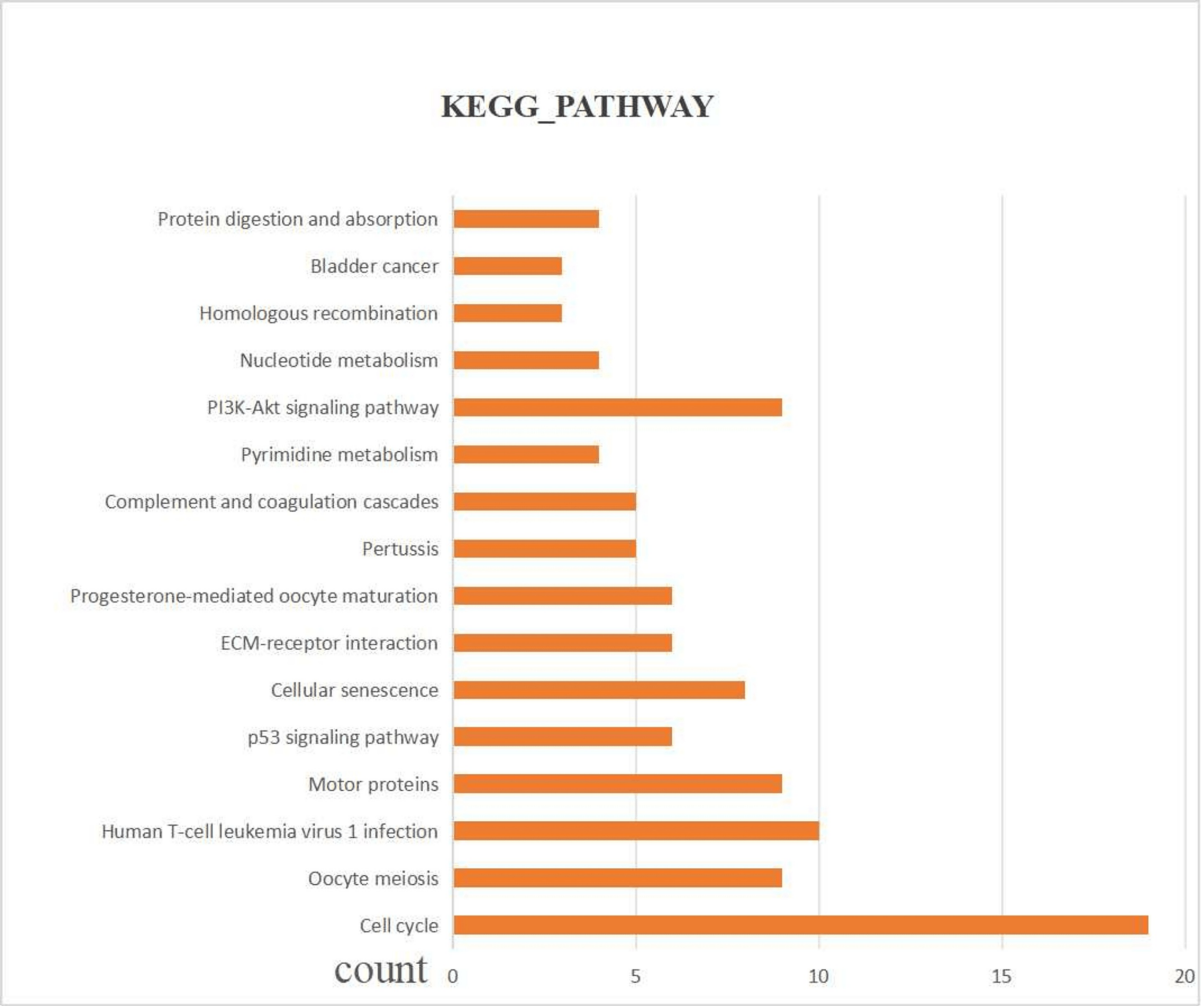
KEGG pathway analysis of TRDEGs in these centers KEGG: Kyoto Encyclopedia of Genes and Genomes, TRDEGs: Telomere-related differentially expressed genes

Through GO enrichment analysis, we identified terms with a false discovery rate (FDR) < 0.05 as significantly enriched. We visualized the top eight terms for cellular component (CC), molecular function (MF), and biological process (BP) categories. The BP terms predominantly included cell division, chromosome segregation, mitotic spindle organization, mitotic spindle assembly checkpoint, mitotic cell cycle, mitotic cytokinesis, mitotic metaphase plate congression, and attachment of mitotic spindle microtubules to kinetochore. The CC terms mainly included kinetochore, spindle, chromosome, centromeric region, microtubule cytoskeleton, mitotic spindle, condensed chromosome outer kinetochore, midbody, centrosome, etc. After filtering with FDR < 0.05, only four terms were included for visualization in the MF category, primarily including microtubule binding, microtubule motor activity, protein binding, and extracellular matrix structural constituents (Figure 3). Similarly, we conducted a KEGG pathway enrichment analysis, which revealed that TRDEGs are mainly enriched in pathways such as cell cycle, oocyte meiosis, human T-cell leukemia virus 1 infection, motor proteins, p53 signaling pathway, PI3K-Akt signaling pathway, and others (Figure 4).

A PPI analysis of the TRDEGs

**Figure 5:**
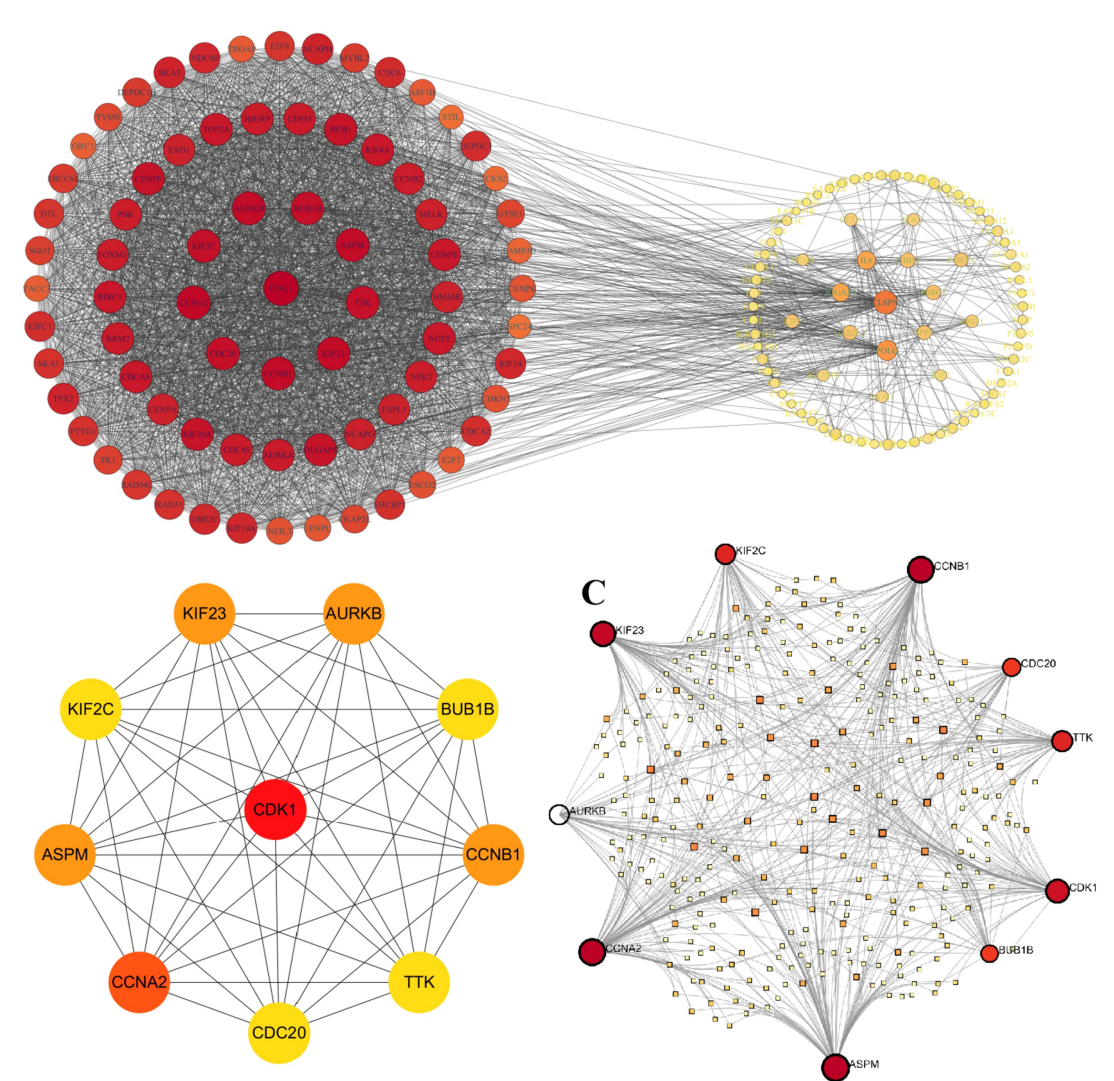
A PPI analysis of the TRDEGs A: The PPI analysis of TRDEGs; B: The top 10 hub TRDEGs; C: The mRNA-miRNA interactions network. The larger circles represent stronger interactions. PPI: Protein-protein interaction, TRDEGs: Telomere-related differentially expressed genes, miRNA: microRNA

**Figure 6:**
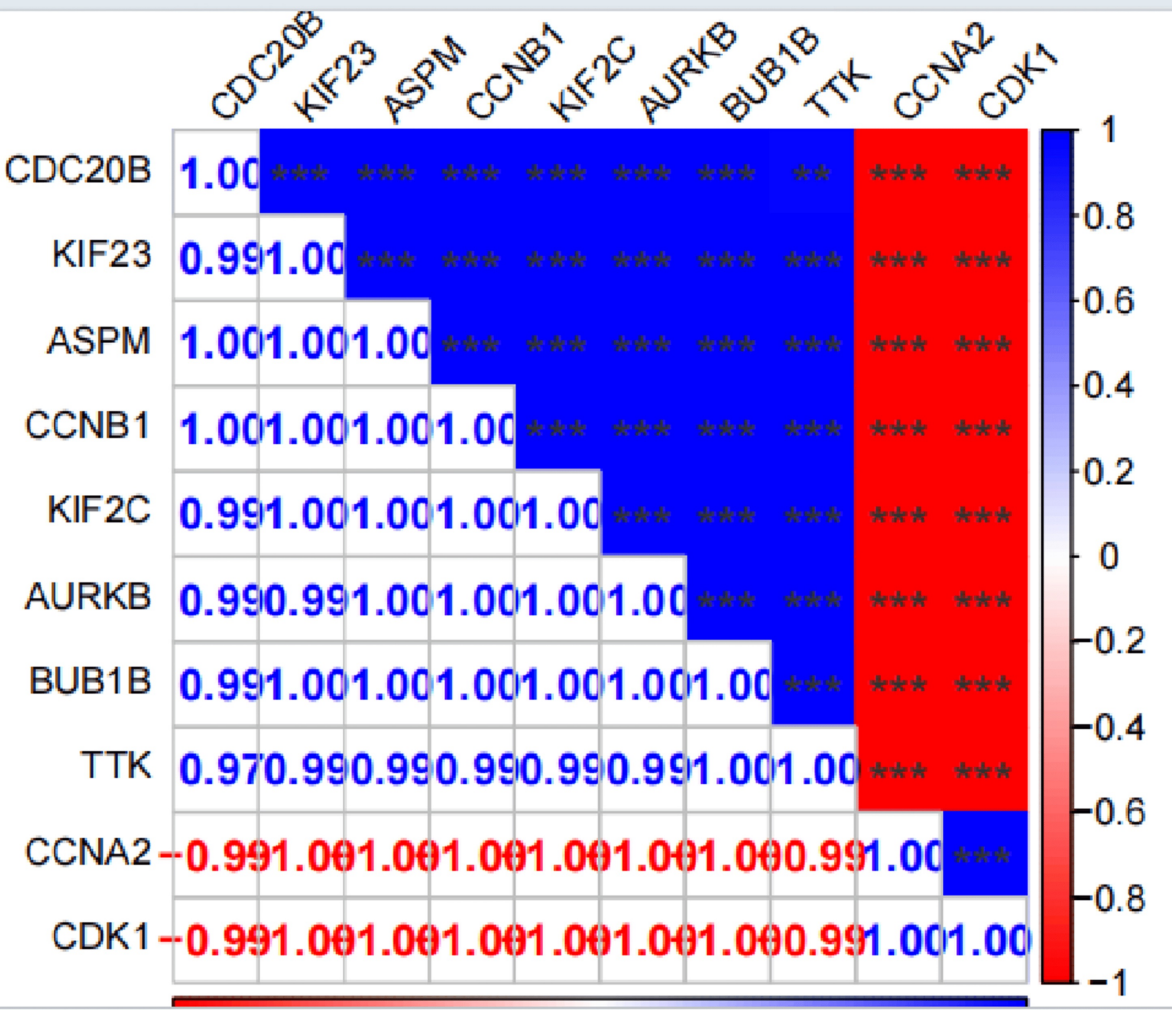
Correlation analysis among the top 10 hub FRDEGs by Pearson analysis FRDEGs: Ferroptosis-related differentially expressed genes

We performed a PPI analysis of TRDEGs using the DAVID website and visualized the results using the Cytoscape software. Based on degree centrality, the top 10 genes were selected. These genes are shown in Figure 5, where CDK1 and CCNA2 are down-regulated and CCNB1, KIF23, ASPM, AURKB, TTK, BUB1B, KIF2C, and CDC20 are up-regulated. Detailed information on these hub TRDEGs is presented in Table 2. Next, we conducted a Pearson correlation analysis on the 10 hub genes. The results revealed strong correlations among all genes. Specifically, CDK1 and CCNA2 showed negative correlations with the other eight genes, while the remaining eight genes exhibited positive correlations with each other, as depicted in Figure 6.

**Table 2:** Top 10 in PPI ranked by the degree method PPI: Protein-protein interaction, TRDEG: Telomere-related differentially expressed gene

Name of TRDEG	Degree
CDK1	85
CCNA2	82
CCNB1	81
KIF23	81
ASPM	81
AURKB	81
TTK	80
BUB1B	80
KIF2C	80
CDC20	80

Validation of hub TRDEGs in the GSE124272 dataset

**Figure 7:**
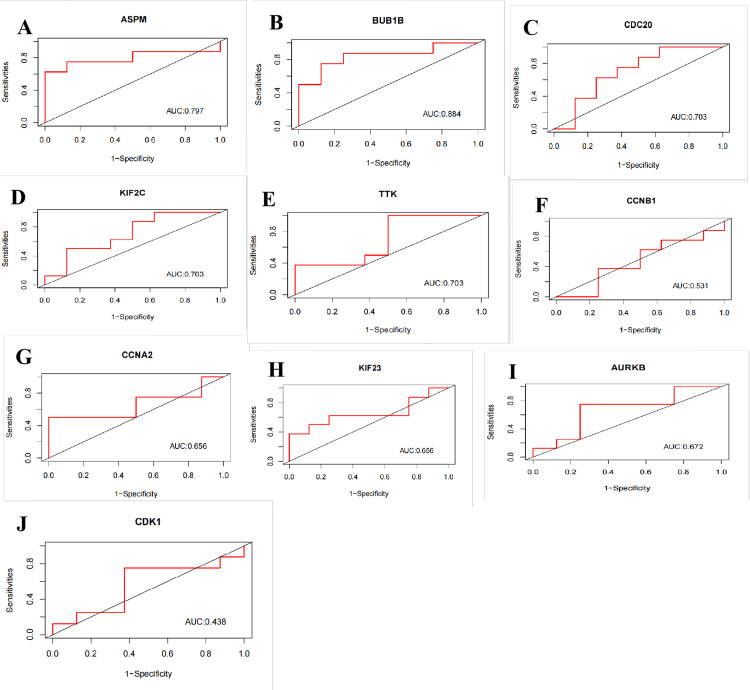
Validation of these hub TRDEGs using the GSE245147 dataset A-I: The ROC analysis of the 10 hub TRDEGs, including ASPM, BUB1B, CDC20, KIF2C, TTK, CCNB1, CCNA2, KIF23, AURKB, and CDK1. The x-axis represents the (1-specificity) and the y-axis represents the sensitivity. TRDEGs: Telomere-related differentially expressed genes, ROC: Receiver operating characteristic

To evaluate the predictive value of these hub FRDEGs, we performed ROC analysis based on the GSE124272 dataset. The ROC curve revealed that the AUC values of six hub genes are greater than or equal to 0.70, including ASPM, BUB1B, CDC20, KIF2C, and TTK. The above data suggests that these five hub TRDEGs have potential predictive values for IVDD (Figure 7).

An mRNA-miRNA interaction analysis on these 10 hub genes

We performed an mRNA-miRNA interaction analysis on the 10 hub genes using the NetworkAnalyst website (Figure 5). The results indicate that hsa-mir-16-5p, hsa-mir-107, hsa-mir-147a, hsa-mir-124-3p, hsa-mir-130a-3p, hsa-mir-195-5p, and hsa-mir-129-2-3p are potentially key miRNAs involved in the interaction with these 10 hub genes (Table 3).

**Table 3:** Top seven in mRNA-miRNA network ranked by the degree method miRNA: microRNA

Entrez ID	Label	Degree	Betweenness
259266	ASPM	104	14278.04
890	CCNA2	96	12457.44
891	CCNB1	94	12322.44
9493	KIF23	80	10900.98
983	CDK1	68	7271.93
7272	TTK	49	4904.76
11004	KIF2C	45	3473.71
9212	AURKB	43	2595.71
991	CDC20	33	1985.69
701	BUB1B	30	1409.29
MIMAT0000069	hsa-mir-16-5p	10	1154.62
MIMAT0000104	hsa-mir-107	10	1154.62
MIMAT0000251	hsa-mir-147a	10	1154.62
MIMAT0000422	hsa-mir-124-3p	10	1154.62
MIMAT0000425	hsa-mir-130a-3p	10	1154.62
MIMAT0000461	hsa-mir-195-5p	10	1154.62
MIMAT0004605	hsa-mir-129-2-3p	10	1154.62

## Discussion

In this study, we performed a comprehensive analysis of TRDEGs to investigate their roles in cellular processes and signaling pathways, in particular their potential implications in diseases such as IVDD. Using a variety of bioinformatic approaches, we uncovered the critical roles of these genes in cell cycle regulation, mitotic processes, and cellular signaling and compared our findings with the existing literature.

Through differential expression analysis, we identified 199 TRDEGs significantly enriched in key biological processes such as cell division, chromosome segregation, and mitotic spindle organization. These processes are crucial for maintaining cellular function and genome stability and are implicated in the pathogenesis of various diseases, including IVDD [16]. Our findings are consistent with previous studies indicating that hub genes such as CDK1, CCNA2, CCNB1, KIF23, ASPM, AURKB, TTK, BUB1B, KIF2C, and CDC20 play critical roles in regulating the cell cycle, mitotic processes, and cell proliferation. Shen et al. demonstrated that CDK1 is overexpressed in most tumor tissues, especially in gastrointestinal tumors, and can regulate the cell cycle [17]. Luo et al. found that the CCNA2 and CCNB1 genes play important roles in cell proliferation in rats by transcriptome analysis [18]. Previous studies suggest that CDK1, CCNA2, and CCNB1 may be associated with inflammation, immune system disorders, and tongue tumors [19]. The KIF23 overexpression accelerates the malignant phenotypes of CC cells and inhibits pyroptosis activation, which is blocked by nigericin treatment. Therefore, KIF23 may play an oncogenic role in CC progression via inhibition of the NLRP3-mediated pyroptosis pathway [20]. Xue et al. found that ASPM is associated with tumors, inflammation, and necrosis [21]. Similarly, Maksiutenko et al. found that CDC20 is closely associated with cell cycle regulation [22]. All of these results suggest that dysregulation of these genes is closely associated with disease development and prognosis, including IVDD. While specific studies on TRGs in IVDD are relatively limited, research suggests that genes involved in cell proliferation and chromosome segregation, such as AURKB and TTK, may play crucial regulatory roles in IVDD pathogenesis [23]. These findings further support our discovery of the importance of these genes in disease mechanisms, providing strong support for their potential as therapeutic targets in clinical applications. Although the sample size in this study is limited, particularly in dataset GSE245147, which may impact the reliability of statistical analyses, our preliminary findings provide a foundation for future larger-scale studies. We recommend increasing sample sizes in future research to further validate these results and enhance the generalizability of the findings.

Our GO enrichment analysis revealed significant enrichment of TRDEGs in CC and MF. Cellular components such as kinetochore, spindle, and centrosome were prominently enriched in the CC category, consistent with previous studies [24,25]. These structures play critical roles in mitosis and are essential for normal cell division and chromosome segregation. In the MF category, functions related to microtubule binding and protein interactions were enriched, further emphasizing the importance of these genes in mitosis and cell cycle regulation [26]. The KEGG pathway analysis further confirmed the enrichment of TRDEGs in pathways such as cell cycle regulation and cancer signaling pathways. These pathways play crucial roles in the development of various diseases, including cancer and IVDD [21,27]. Previous research indicates that aberrant activation or inhibition of these pathways is closely associated with cell proliferation, metastasis, and treatment resistance [28].

Protein-protein interaction network analysis revealed complex interactions among hub genes, encompassing not only cell cycle regulation but also biological processes such as cell proliferation, signal transduction, and cell death. Additionally, mRNA-miRNA interaction analysis identified key miRNAs such as hsa-mir-16-5p, hsa-mir-107, and hsa-mir-147a, which may regulate the expression levels of these hub genes, thereby influencing intervertebral disc cell function and disease progression. This is the same as previous studies, corroborating that there is a regulatory relationship between the appeal miRNAs and the hub genes that we can obtain [29,30]. The identified hub genes have potential as early diagnostic biomarkers or therapeutic targets for IVDD. Further research should assess their applicability in clinical settings and explore their potential role in developing novel therapeutic strategies.

Our study not only deepens our understanding of TRGs in cellular biological processes but also provides a theoretical basis for identifying and developing new therapeutic targets. Understanding the critical roles of these genes in cell cycle regulation, mitosis, and genome stability holds significant clinical implications for the treatment and prevention of diseases such as cancer and IVDD. To further elucidate the roles of TRGs in IVDD and other diseases, future research could focus on several aspects: validating specific TRDEGs in IVDD pathogenesis using animal models, exploring potential therapeutic targets based on these genes, such as small molecule drugs or gene editing technologies, and integrating clinical sample data analysis to assess the potential of these genes as biomarkers for IVDD. Intervertebral disc degeneration is a multifactorial process, and in addition to TRGs, other molecular mechanisms, such as inflammation and extracellular matrix degradation, play crucial roles in disease pathology. Future studies should explore these pathways to gain a more holistic understanding of IVDD.

This study has several limitations. First, the relatively small sample size, particularly three degenerated and three non-degenerated NP tissue samples, limits the statistical power and generalizability of the findings. A larger, more diverse sample set is necessary for more robust conclusions. Second, while bioinformatics analyses identified key TRGs, no experimental validation, such as qPCR or Western blot, was performed to confirm these findings. This limits the direct applicability of the results. In future work, qPCR and Western blot experiments will be incorporated to confirm the differential expression of the identified genes, strengthening the clinical applicability of the findings. Third, the study's focus on TRGs may overlook other crucial molecular pathways involved in IVDD, such as inflammation and extracellular matrix degradation. These pathways should be explored in future research to provide a more comprehensive understanding of disease pathogenesis. While this study relies primarily on bioinformatics tools, we recognize the limitations of these methods, such as the potential for false positives or the omission of biologically significant interactions. We have therefore interpreted the results with caution and emphasized the need for further validation.

## Conclusions

Our study provides a comprehensive analysis of TRGs in IVDD, identifying key regulatory molecules and pathways involved in the disease process. The findings offer new insights into the molecular mechanisms of IVDD and highlight potential biomarkers and therapeutic targets. Continued research in this area will be crucial for advancing our understanding and management of IVDD.
